# Neonatal consequences of maternal exposure to the chikungunya virus: Case reports

**DOI:** 10.1097/MD.0000000000025695

**Published:** 2021-04-30

**Authors:** Thamirys Cosmo Grillo Fajardo, Rosa Estela Gazeta, Daniel Thome Catalan, Alexandra Siqueira Mello, Andrea Cristina Botelho da Silva, Ana Paula Antunes Pascalicchio Bertozzi, Geovane Ribeiro Dos Santos, Clóvis Antonio Lopes Pinto, Cairo Oliveira Monteiro, Rafael Rahal Guaragna Machado, Danielle Bruna Leal Oliveira, Edison Luiz Durigon, Saulo Duarte Passos

**Affiliations:** a Laboratory of Paediatric Infectology, Jundiaí School of Medicine; b Department of Paediatric, Jundiaí School of Medicine; c Department of Morphology and Pathology, Jundiaí School of Medicine; d Pathology Department, AC Camargo Cancer Center; e Department of Microbiology, Institute of Biomedical Sciences, University of Sao Paulo, Sao Paulo, SP, Brazil.

**Keywords:** arbovirus infections, case report, chikungunya virus, cohort study, new-born

## Abstract

**Rationale:**

The chikungunya virus (CHIKV) was first isolated in a Tanzanian epidemic area between 1952 and 1953. The best description of the CHIKV transmission during pregnancy can be found in a well-documented epidemic in 2005, in the “La Reunion” island, a French territory located in the Indian Ocean, in which about one-third of the population was infected. Reports of arbovirus infections in pregnancy are increasing over time, but the spectrum of clinical findings remains an incognita among researchers, including CHIKV.

**Patient concerns:**

In this report, it was possible to verify 2 cases exposed to CHIKV during foetal period and the possible implications of the infection on gestational structures and exposed children after the birth.

**Diagnosis:**

In both cases, the mothers were positive by laboratory tests in serologic analysis for CHIKV, as ezyme-linked immunossorbent assay (ELISA), plaque reduction neutralisation testing (PRNT) and immunofluorescence (IF); but there were no positive tests in quantitative polymerase chain reaction (qPCR) for mothers or children.

**Interventions:**

The exposed children were followed up in a paediatrics clinic in order not only to provide the medical assistance, but also to verify child development and the possible implications and neurocognitive changes caused by gestational infection.

**Outcomes:**

There were neurological and developmental changes in one of the children followed up on an outpatient basis. There was an improvement in the neurological situation and symptoms only 3 years and 1 month after birth.

**Lessons:**

Based on the cases presented, we can conclude that clinical symptoms of CHIKV maternal infection may occur late in new-borns and can affect their development.

## 1 Introduction

The chikungunya virus (CHIKV, *Togaviridae* family, *Alphavirus* genus) is an RNA virus, whose transmission occurs through insect bites, especially Aedes Aegypti and Aedes Albopictus, which are also involved in other arbovirus transmissions, such as Zika virus (ZIKV) and dengue virus (DENV).^[1–3]^

CHIKV was first isolated in a Tanzanian epidemic area between 1952 and 1953. It is named after a Makonde word that means “that which bends up” due to the severe articular pain that the patients present with, when symptomatic.^[4,5]^ Currently, CHIKV is an emergent disease that is universally distributed. The best description of the CHIKV transmission during pregnancy can be found in a well-documented epidemic in 2005, in the “La Reunion” island, a French territory located in the Indian Ocean, in which about one-third of the population was infected.^[6]^

Since then, there has been an increase in reports about vertical CHIKV transmission, indicating that the foetus of the pregnant infected mother can present the disease up to the 4th day of postnatal life. In these cases, the literature shows a possibility of 50% of vertical transmission, with cognitive consequences to the new-born, but only few reports describe the clinical spectrum of the disease in new-borns and their follow-up.^[6–10]^

In this paper, we describe 2 cases of Zika Cohort Jundiai that were followed by clinical and laboratory examinations during pregnancy and postnatally.

## 2 Patient information

### 2.1 Case 1

TSM (G1) was a 23-year-old female who presented in her second pregnancy with no previous abortions. The patient underwent prenatal examinations and, at the end of the third trimester of gestation, presented with mild myalgia, arthralgia, and headaches, followed by moderate neurological symptoms (inferior limb motor weakness and impaired deambulation) and fever.

RSSS (R1), female, was born by vaginal delivery at 38 weeks and 4 days of gestational age, weight 3280 g (IG standard score [zs] +0.53), length 48 cm (IG zs −0.24), head circumference 31 cm (IG zs −1.99), and Apgar 8/9. TORCH (Toxoplasma, Rubella, Cytomegalovirus and Herpes simplex virus I and II infection) was negative, cytomegalovirus quantitative polymerase chain reaction (qPCR) was negative, and the urine and blood samples were both normal in laboratory analysis. All screening new-born exams were normal, and she was discharged after 3 days.

At delivery, placental biopsies were performed, and anatomopathological examination showed intervillous fibrin deposits, intervillous calcification, and infarction areas.

The mother's peripheric blood was tested for CHIKV, ZIKV, DENV (Euroimmun) and for TORCH (Sérion), using the ezyme-linked immunossorbent assay (ELISA) method, according to the manufacturer's recommendations. The mother's blood was positive for CHIKV for both antibodies, anti IgG immunoglobulin (IgG) and anti IgM immunoglobulin (IgM). This result was later confirmed by plaque reduction neutralisation testing (PRNT), utilising the same sample (positive: 90 > 20), and by IF (immunofluorescence). Serum was also tested for CHIKV by qPCR, with a negative result.

**Figure 1:**
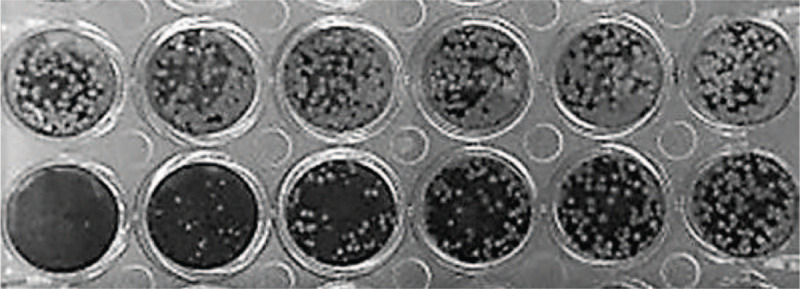
PRNT test of R1 in line 1, in horizontal position, and PRNT test of G1 in line 2, in horizontal position.

The R1 urine sample was tested for CHIKV by qPCR and was negative. Laboratory examinations at 1 month of age were negative (see Table 1 and Fig. 1). R1 was clinically followed-up. At 41 days of age, she presented with maculopapular exanthema. Neurological examination results were normal. At 83 days of age, R1 presented with axial hypotonia and altered plantar support, and could not track moving objects with her eyes. At 132 days of age, opisthotonos was observed. During the 6th month of age, she was not able to sit, grab objects, or babble. These neurological alterations slowly improved by 9 months of age, except for the speech abilities (she did not present lallation and was not able to repeat syllables or babble).

**Table 1:** G1 and R1 laboratory results.

	Period	Exam	Method	Material	Result
G1	Delivery	CHIKV IgG	ELISA	Serum	Positive
		CHIKV IgM	ELISA^∗^	Serum	Positive
		CHIKV IgG	IF^†^	Serum	Positive
		CHIKV IgM	IF^†^	Serum	Positive
		CHIKV	PRNT^‡^	Serum	Positive (90 > 20)
		CHIKV	qPCR^§^	Serum	Negative
R1	Birth	CHIKV	qPCR^§^	Urine	Negative
R1	One month	CHIKV IgG	ELISA^∗^	Serum	Negative
		CHIKV IgM	ELISA^∗^	Serum	Negative
		CHIKV	PRNT^‡^	Serum	Negative

At 1 year of age, she was able to sit and crawl, but she could not repeat words. Cognitive Bayesian evaluation was normal. During the last follow-up, at 3 years and 1 month of age, neurological examination was normal (see Table 2).

**Table 2:** R1 clinical findings.

Days of life	Clinical findings
41	• Maculopapular exanthema
83	• Axial hypotonia• Altered plantar support• Impossibility of tracking moving objects
132	• Pathological posture• Hands persistently closed• Altered plantar support
181	• Hands persistently closed• Impossibility to seat, babble
279	• Uncapable to babble or repeat syllables
363	• Voluntary hand movements• Seats and crawls• Uncapable to repeat words

### 2.2 Case 2

DMB (G2) was a 23-year-old female who presented in her second pregnancy with no previous abortions. The patient underwent prenatal examinations, and during the first trimester of pregnancy, the patient presented with severe myalgia, headache, arthralgia, articular oedema, pruritus, and neurologic symptoms. At that time, she resided in northeast Brazil. She was positive for ZIKV by qPCR. At delivery, as a Zika Cohort Jundiai patient, she presented negative on ZIKV serology.

LBA (R2), a new-born female, was born by caesarean section at 40 weeks and 3 days of gestational age. Birth weight was 4205 g (gestational age [IG] standard score [zs] +2.0), length 52 cm (IG zs +1.6), and head circumference was 37 cm (IG zs+ 2.6). Initial physical examination was normal, head computed tomography scan and new-born screening exams were all normal.

At delivery, samples were collected from G2 and R2 for analysis: peripheral blood, cord blood, urine, and placenta. Placental pathology showed perivillous fibrin deposits and calcifications. G2 and R2 blood samples were tested for CHIKV, ZIKV, DENV (Euroimmun) and for TORCH (Sérion), using the ELISA method, according to the manufacturer's recommendations.

G2 tests showed IgG antibodies to DENV and CHIKV, and IgM antibodies to CHIKV. These CHIKV results were recently confirmed by PRNT (Positive: 90 > 20) and IF. The qPCR for CHIKV was negative. The R2 blood cord was IgG-positive for both DENV and CHIKV. R2 urine samples were tested by qPCR for ZIKV and CHIKV, and they were both negative. Sequentially, R2 samples were collected at 1 month of age, and CHIKV ELISA was IgG-positive (IgM-negative) and PRNT was positive (90 > 20).

**Figure 2:**
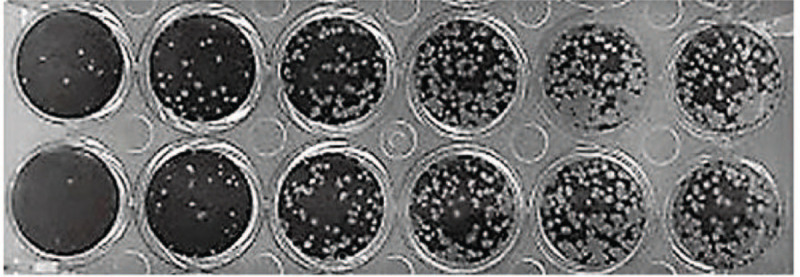
PRNT test of R2 in line 1, in horizontal position, and PRNT test of G2 in line 2, in horizontal position.

During the short period of follow-up (only up to 1 month of age), we could not find any neurological alteration. G2 and R2 were then lost to follow-up (see Table 3 and Fig. 2).

**Table 3:** G2 and R2 laboratory results.

	Time	Exam	Method	Material	Result
G2	Delivery	CHIKV IgG	ELISA^∗^	Serum	Positive
		CHIKV IgM	ELISA^∗^	Serum	Positive
		CHIKV IgG	IF^†^	Serum	Positive
		CHIKV IgM	IF^†^	Serum	Positive
		CHIKV	PRNT^‡^	Serum	Positive (90 > 20)
		CHIKV	qPCR^§^	Serum	Negative
R 2	Birth	CHIKV IgG	ELISA^∗^	Blood cord	Positive
		CHIKV IgM	ELISA^∗^	Blood cord	Negtive
		CHIKV	qPCR^§^	Urine	Negtive
R 2	One month	CHIKV IgG	ELISA^∗^	Serum	Positive
		CHIKV IgM	ELISA^∗^	Serum	Negative
		CHIKV	PRNT^‡^	Serum	Positive (90 > 20)

## 3 Discussion

Approximately 90% of the pregnancies occur in areas susceptible to arbovirus infections, and most of them occur in underdeveloped areas, making the diagnosis even more difficult.^[11]^ Reports of arbovirus infections in pregnancy are increasing over time, but the spectrum of clinical findings remains an incognita among researchers, including CHIKV.^[12,13]^

Despite the fact that, in both cases, we could not observe CHIKV genetic material in the pregnant mothers’ peripheral blood or in new-borns’ urine, we could detect maternal IgG and IgM antibodies by ELISA test, IF, and PRNT, indicating that the mothers were in the convalescent phase. The absence of viremia detected by qPCR prevented the laboratory diagnosis of vertical transmission in both cases. Nevertheless, if we look at the clinical findings presented by R1, we could remotely consider the possibility of this transmission, which could not be confirmed in the laboratory. We could infer the possibility of a window period in R1 once this baby manifested the symptoms later.

Considering the laboratory findings, we could not demonstrate active infection in both new-borns, but R1 exhibited possible consequences of the maternal CHIKV exposure: the exanthema, the neurological, and non-permanent alterations (that lasted for about 1 year). This finding is in accordance with other studies that showed a minor interference of CHIKV in the foetus, but it is conflicting with findings during the neonatal period, which demonstrate clinical findings between the third and seventh days after birth.^[6,8,14]^

In both cases, the new-borns did not require any extra care or treatment. They did not present signs of sepsis, respiratory distress, or hemodynamic instability, confirming a mild presentation. The literature points to severe manifestations related to vertical transmission, even increased risk of death and increased mortality rate.^[15]^ Data in our study can be explained by the placental barrier, which may have acted as a protection to the foetus.

Many studies have confirmed the efficacy of placenta in protecting the foetus against viral agents, such as CHIKV.^[6,16]^ At the same time, it is known that CHIKV can cause damage to placental tissue, altering its physiology, oxygen exchange, and nutrient transfer. This altered physiology may be directly responsible for foetal development, leading to intrauterine growth restriction (IUGR).^[6,17,18]^ In our cases, we observed a placental involvement (fibrin deposits in the intervillous spaces and calcifications).

We believe that R1 may have presented with IUGR, especially when we consider the head circumference (zs -1.99), and the neurological findings during follow-up, which were all normalising over time.

Based on the cases presented, we conclude that despite the fact that the viremia was not observed at the time of delivery, we could not exclude the possibility of vertical transmission, as G1 and G2 had a well-documented CHIKV infection by more than 1 laboratory diagnostic technique.

Additionally, maternal CHIKV infection in the third trimester may have negative consequences on the placenta, leading to foetal IUGR and decreased foetal growth during this period.

Clinical signs and symptoms of CHIKV maternal infection may occur late in the new-born, and can, secondarily affect the expected development of these babies. It is important to emphasise the correct diagnosis to promote the best follow-up of these new-borns.

## Acknowledgments

The authors would like to acknowledge all the patients who participated in this study, as well as all the volunteers, researchers, and professionals involved in this project named “Cohort Zika Jundiaí Consortium”. We would like to acknowledge also all the researchers in the Laboratory of Clinical and Molecular Virology, Institute of Biomedical Sciences, University of Sao Paulo and the researchers in the Laboratory of Paediatric Infectiology, Jundiaí, especially thank Isabella Baden Ferreira and Priscila de Oliveira Costa Santos.

We also would like to thank Editage (www.editage.com) for English language editing.

## Author contributions

**Conceptualization:** Ana Paula Antunes Pascalicchio Bertozzi, Daniel Thome Catalan, Saulo Duarte Passos, Thamirys Cosmo Grillo Fajardo.

**Investigation:** Thamirys Cosmo Grillo Fajardo, Rosa Estela Gazeta, Daniel Thome Catalan, Alexandra Siqueira Mello, Andrea Cristina Botelho Silva, Ana Paula Antunes Pascalicchio Bertozzi, Geovane Ribeiro dos Santos, Clovis Antonio Lopes Pinto, Cairo Oliveira Monteiro, Rafael Rahal Guaragna Machado, Danielle Bruna Leal Oliveira, Edson Luiz Durigon, Saulo Duarte Passos.

**Project administration:** Saulo Duarte Passos, The Jundiai Zika Cohort Group.

**Supervision:** Daniel Thome Catalan, Ana Paula Antunes Pascalicchio Bertozzi, Saulo Duarte Passos.

**Validation:** Daniel Thome Catalan, Ana Paula Antunes Pascalicchio Bertozzi, Saulo Duarte Passos.

**Visualization:** Daniel Thome Catalan, Ana Paula Antunes Pascalicchio Bertozzi, Saulo Duarte Passos.

**Writing – original draft:** Thamirys Cosmo Grillo Fajardo.

**Writing – review & editing:** Daniel Thome Catalan, Ana Paula Antunes Pascalicchio Bertozzi, Saulo Duarte Passos.
